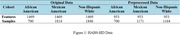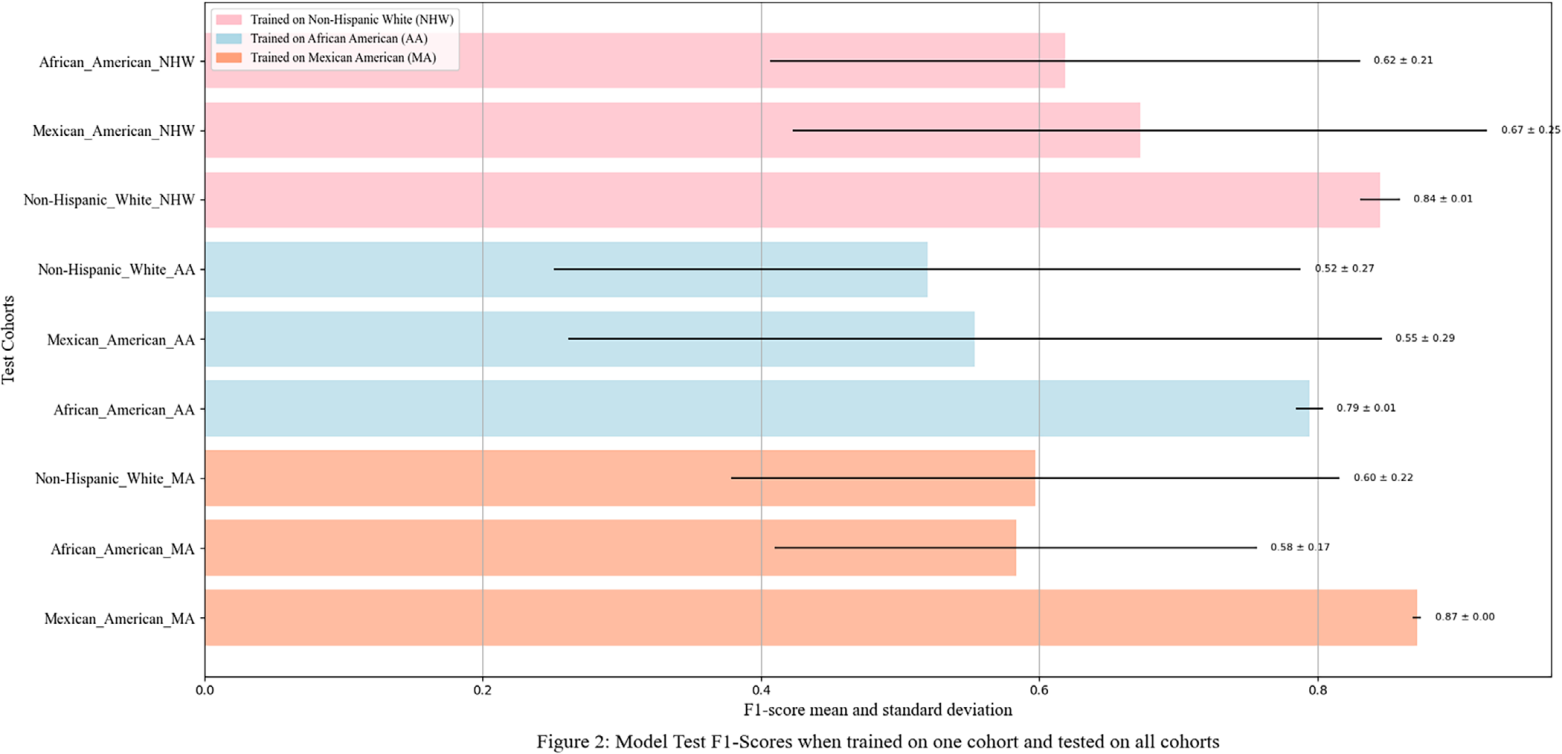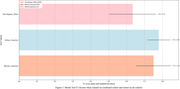# A Systematic Analysis on Clinical Diagnosis of Alzheimer’s Disease Using HABS‐HD Data

**DOI:** 10.1002/alz.095595

**Published:** 2025-01-09

**Authors:** Neha Goud Baddam, Bizhan AlipourPijani, Serdar Bozdag

**Affiliations:** ^1^ University of North Texas, Denton, TX USA; ^2^ BioDiscovery Institute / University of North Texas, Denton, TX USA

## Abstract

**Background:**

Alzheimer’s Disease (AD), a prevalent neurodegenerative condition, poses a significant global health challenge, with a projected increase in cases. Existing datasets often lack representation from certain racial groups, raising concerns about bias in AD diagnosis models. We hypothesize that a Machine Learning (ML) model trained on one racial group performs well on that group but struggles to predict cases in a different racial group. To explore this hypothesis, we leverage the Health & Aging Brain Study ‐ Health Disparities (HABS‐HD) dataset to predict AD diagnosis.

**Method:**

The HABS‐HD encompasses Non‐Hispanic White, Mexican American, and African American cohorts (Figure 1) with 1469 features and 4360 samples. We addressed a 31.88% missing data rate by employing K‐Nearest Neighbor and Iterative imputation techniques. As for feature selection, we retained features with a standard deviation between 0.02 and 100, then applied several feature selection methods such as Lasso, Boruta, Genetic selection, Recursive Feature Elimination, and Meta‐transformer. Initially, we applied these methods to each cohort to show distinct influences, followed by their application to the combined data to reveal collective insights. We trained several ML models using training data from individual cohorts and combined data. We stratified test‐train splits and used balanced models to ensure unbiased training.

**Result:**

We show the result of Random Forest model, as it outperformed other ML models. When the model is trained on one cohort (e.g., African American) performs well in that cohort with low standard deviation, while it performs poorly in other cohorts (e.g., Non‐Hispanic White, Mexican American) with high standard deviation (Figure 2), confirming our hypothesis that training within a cohort excels but may struggle across others. The prediction performance was increased when the models trained on combined data were applied on these cohorts, however the model performance for the original cohort was higher when it was trained with its own features.

**Conclusion:**

This study emphasizes the importance of racial diversity in datasets for accurate predictions and contributes to designing fair AD prediction models. Our results suggest that ML models need to be trained on racially diverse cohorts to gain higher and more fair performance.